# A mechanistic evaluation of the angiogenic properties of a dehydrated amnion chorion membrane in vitro and in vivo

**DOI:** 10.1111/wrr.12757

**Published:** 2019-08-29

**Authors:** John P. McQuilling, Miranda Burnette, Kelly A. Kimmerling, MaryRose Kammer, Katie C. Mowry

**Affiliations:** ^1^ Research and Development, Organogenesis 2641 Rock Ridge Lane Birmingham Alabama 35216

## Abstract

Angiogenesis is essential for the successful repair of tissues; however, in many chronic conditions, angiogenesis is inhibited. Placental tissues have been shown to illicit an angiogenic response both in vitro and in vivo, and the angiogenic properties of these tissues likely contribute to observed clinical outcomes. Although there is some work describing the angiogenic effects of these tissues, comparatively little has been done to determine the possible mechanisms responsible for this effect. The purpose of this study was to conduct a thorough evaluation of a commercially available dehydrated amnion chorion membrane to better understand how these tissues may promote angiogenesis. The proteomic content of this tissue was evaluated using a high throughput proteomic microarray, and then the effects of these grafts were evaluated in vivo using subcutaneous gelfoam sponge implants containing conditioned media (CM) from the graft. Human microvascular endothelial cells were then used to determine how released factors effect migration, proliferation, gene expression, and protein production in vitro. Finally, to elucidate potential signaling‐pathways through which tissue‐derived factors act to induce pro‐angiogenetic phenotypes in endothelial cells in vitro, we performed a global analysis of both serine/threonine and tyrosine kinase activity. Kinomic and proteomic data were then combined to generate protein–protein interaction networks that enabled the identification of multiple growth factors and cytokines with both pro‐ and anti‐angiogenetic properties. In vivo, the addition of CM resulted in increased CD31 and αSMA staining and increases in pro‐angiogenic gene expression. In vitro, CM resulted in significant increases in endothelial proliferation, migration, and the expression of granulocyte‐macrophage colony‐stimulating factor, hepatocyte growth factor, and transforming growth factor beta‐3. Integrated kinomic analysis implicated ERK1/2 signaling as the primary pathway activated following culture of endothelial cells with dehydrated amnion/chorion membrane (dACM) CM. In conclusion, dACM grafts triggered pro‐angiogenic responses both in vitro and in vivo that are likely at least partially mediated by ERK1/2 signaling.

## INTRODUCTION

Angiogenesis, the development of new blood vessels from existing ones, is an essential part of tissue repair. Regardless of the type of tissue undergoing angiogenesis, the process retains many characteristics. Following the disruption of the preexisting vascular network through injury and consequent formation of a hematoma, the initial inflammatory response results in the production of angiogenic growth factors, including TGFβ‐1, TGF‐α, FGF, and VEGF.^1^ The release of these growth factors in combination with localized hypoxia results in the initiation of sprouting angiogenesis from preexisting vessels into a fibrin or fibrinogen rich clot. As this process continues, endothelial cells degrade the preexisting matrix, and migrate and proliferate toward the site of injury. These sprouts will anastomose to form a network, which over time is stabilized by the recruitment of pericytes and vascular smooth muscle cells through several pathways involving platelet‐derived growth factor BB (PDGF‐BB), sphingosine‐1‐phospate‐1 (S1P1), and angiopoietin‐1 (Ang1).^2^ As this network matures, additional pruning and remodeling occurs to improve the overall organization of the network. Pruning of the vascular network by the expression of antiangiogenic growth factors such as Sprouty2 and pigment epithelium‐derived factor (PEDF) leads to a more organized network.^3^

Disease states and comorbidities, such as diabetes, age, and obesity, can often inhibit angiogenesis, stalling tissue repair and ultimately leading to chronic injuries. Abnormal angiogenesis in diabetes is well established and is one cause of delayed wound healing in multiple tissues types^4^; aging can also adversely affect angiogenesis^5^ and detrimentally impact tissue repair.^6^ Additional strategies are often required for overcoming angiogenic dysfunction in these patient populations.^3^

Placental‐derived tissues have been shown to support repair of several tissue types, including bone,^7^ tendon,^8^ and skin.^9^ They also have known angiogenic properties, often attributed to the presence of angiogenic growth factors within the matrix.^10^, ^11^, ^12^ Although numerous studies have shown angiogenic properties of placental tissues in a variety of models, relatively little work has been done to characterize the potential mechanisms through which these tissues may promote angiogenesis. Consequently, the purpose of this study is to both study the angiogenic properties of a commercially available dehydrated amnion/chorion membrane (dACM) in vitro and in vivo, as well as identify the growth factors and intracellular signaling pathways that may be responsible for its effects.

## METHODS

### Dehydrated amnion chorion membrane

dACM (NuShield, Organogenesis Canton MA) is a sterilized dehydrated placental‐derived graft consisting of both the amnion and chorion layers of the placental membrane. Placentas were donated with informed consent after planned cesarean sections, and all processing was completed in accordance with the Food and Drug Administration's Good Tissue Practices (GTP) and American Association of Tissue Banks (AATB) standards. All donors were screened for medical issues, social issues, and communicable diseases that could affect donor suitability.

### Proteomic evaluation of dACM

Proteomic evaluation of dACM grafts was conducted using the Quantibody® Human Kiloplex Array (RayBioTech, Norcross, GA). This array is a multiplexed sandwich ELISA‐based quantitative array platform with the ability to detect 1,000 human proteins. For this evaluation, a total of 10 dACM grafts from separate human donors were used. Protein extraction and quantification was conducted independently by the manufacturer of the array. dACM was minced and incubated in lysis buffer (Cat #: AA‐LYS‐10 mL: RayBioTech) at a ratio of 1 mL buffer to 35 mg tissue overnight at 4 °C. Following incubation, grafts were homogenized, centrifuged, and the supernatant was collected and used for analysis. Following quantification by the manufacturer, data were further analyzed to focus on proteins that were, on average, above the level of detection across the 10 donors. These proteins were annotated with Uniprot^13^ keywords and collated to determine the most common biological process, molecular function, and cellular component annotations. Gene Set Enrichment Analysis^14^ (Broad Institute, UC San Diego) was run using the pre‐ranked list option with proteins sorted by average abundance. Enriched terms were determined from Gene Ontology gene sets^15^ with 1,000 permutations and a minimum size of three genes. Proteins were also annotated with manually selected Gene Ontology Terms based on UniProt Gene Ontology tags.

### In vivo assessment of dACM

The protocol for this study was reviewed and approved by the Bridge PTS IACUC (protocol 17‐02). Sprague Dawley rats (300–400 g) were purchased from Charles River Labs (Wilmington, MA). Following anesthesia, the skin over the dorsal cervical region was clipped and appropriately disinfected. Two 2‐cm long incisions were made on the midline perpendicular to the spine and absorbable gelatin sponges (Gelfoam®, Pharmacia & Upjohn Company, Kalamazoo, MI) were implanted. For animals in the treatment group, sponges were soaked with conditioned media (CM); CM was generated by incubating dACM in EBM‐2 basal media (ThermoFisher, Waltham MA) at a concentration of 1 cm^2^ per mL media for 5 days at 4 °C. Control groups consisted of gelatin sponges soaked with unconditioned media or sponges soaked with media supplemented with 25 μg/mL heparin and 10 μg/mL recombinant fibroblast growth factor‐2 (FGF‐2) (Peprotech, Rocky Hill, NJ). Wounds were closed with staples and surgical glue and animals were supported with appropriate analgesics.

At 7 and 14 days, implants were retrieved and halved, with one half placed in 10% neutral buffered formalin for histological evaluation and the second half placed in RNAzol (after removal of surrounding connective tissue) and stored at −80 °C. Fixed samples were embedded and sections (5 μm thick) were stained with either CD31 or αSMA with hematoxylin counterstaining. Isotype controls were stained for each implant for both CD31 and αSMA. Stained slides for each implant were imaged and analyzed to determine the percent of area stained for cluster of differentiation 31 (CD31) or alpha smooth muscle actin (αSMA) as an indicator for blood vessel density. For gene expression analysis, three assay media soaked control sponges and three soaked in CM were collected in RNAzol and were evaluated using the TaqMan® Array Rat Angiogenesis 96‐well Plate (ThermoFisher). Each experimental gene was normalized to 18 s and the relative expression of mRNAs was calculated by the 2^−△△Ct^ method.

### In vitro assessment of dACM

#### *Endothelial cell culture*

For proliferation and migration experiments, three independent cell lines of human microvascular endothelial cells (HMVEC) were obtained (Lonza, Walkersville, MD) and expanded based on the manufacturer's instructions. For gene expression and kinomics experiments, two independent cell lines of HMVECs were obtained and cultured per the manufacturer's instructions (Cell Applications, San Diego, CA).

#### *Cell proliferation*

Cell proliferation experiments were conducted as previously described.^12^ Briefly, HMVECs were cultured with 50%, 25%, and 10% (by volume) dACM CM for up to 14 days under standard conditions. Unless otherwise noted, CM was generated by incubating dACM in a low serum assay media at a concentration of 1 cm^2^ per mL media for 5 days at 4 °C. For controls, cells were cultured in either growth media or assay media alone without CM. Cell proliferation was assessed using AlamarBlue (ThermoFisher, Waltham MA) according to the manufacturer's instructions at 3, 7, 10, and 14 days of culture.

#### *Cell migration*

HMVEC migration in response to CM was evaluated using a standard boyden chamber assay. For migration experiments, CM was generated by incubating dACM in a low serum assay media consisting of DMEM:F12 with 2.5% FBS at a concentration of 1 cm^2^ per mL media for 24 to 168 hours at 4 °C. CM was diluted to 50 vol% with fresh assay media before use. Boyden chambers were coated with 5 μg/mL fibronectin (Fisher Scientific, Pittsburgh, PA) overnight at room temperature. Plates were rinsed three times with phosphate buffered saline (PBS). Cells at approximately 80% confluence were serum‐starved overnight, trypsinized and added to the top of the inserts at a concentration of 100,000 cells/well. About 1 mL of either assay media (negative control), assay media +10% FBS (positive control), or CM was added to the bottom reservoir of the plate. Plates were incubated under standard culture conditions for 24 hours. Nonmigrating cells remaining on the top of the inserts were removed using a cotton tip applicator, and cells that had migrated were fixed with 4% paraformaldehyde for 10 minutes prior to staining with a crystal violet solution. Inserts were imaged using an inverted microscope (Nikon, Melville, NY). Representative images were then used to quantify cell migration.

#### *Gene and protein production*

HMVECs were cultured under standard conditions in six‐well plates in assay media with or without 25% CM (by volume). Following 24, 48, and 72 hours of culture, both the supernatant and cell monolayers were collected and stored at −80 °C until use. Total RNA was extracted and quantified from cell monolayers collected in RNAzol, on a NanoDrop 2000 UV/Vis spectrophotometer (Thermo Scientific). The Verso cDNA synthesis kit (ThermoFisher Scientific, Pittsburgh, PA) was utilized for transcription to cDNA and mRNA were measured using TaqMan probes on a Quantstudio 3 PCR System (Applied Biosystems, Waltham, MA). Each experimental gene was normalized to glyceraldehyde 3‐phosphate dehydrogenase (GAPDH) and the relative expression of mRNAs was calculated by the 2^−△△Ct^ method. A complete list of targets is located in the Supporting Information Table S1.

#### *Kinomics and network analysis*

To identify the signaling pathways through which dACM‐derived factors induce the phenotypes observed in this study, we performed a global analysis of both serine/threonine and tyrosine kinase activity using a high‐throughput commercial platform (PamGene).^16^, ^17^ Two lots of HMVECs were brought to ~70% confluence before being serum starved overnight in assay media (MCDB‐131 + 0.25% FBS). The next day, cells were rinsed one time with 5 mL of PBS and treated with either assay media (AM) or 25% dACM CM. To determine the optimal timepoint to run on the kinomics platform, samples were collected after 5 minutes, 15 minutes, 30 minutes, 45 minutes, 1 hour, 2 hours, and 4 hours of treatment by aspirating media, washing cells with 5 mL of cold PBS, and incubating cells for 30 minutes with 1 mL of MPER (Sigma, St. Louis, MO) with 1:100 Halt Protease and Phosphatase Inhibitor Cocktail (ThermoFisher, Waltham MA). Lysate was collected, transferred to pre‐chilled microcentrifuge tubes, and spun at 14,000×*g* for 10 minutes at 4°C before being frozen at −80°C.

Phospho‐panAKT (T308) blots were used to select the 15 and 30‐minute timepoints for full analysis. Lysates from these time points were loaded onto the tyrosine chip (PTK v14.0, 15 ug) and serine–threonine chip (STK v8.0, 2 μg) arrays (PamGene International, Hertogenbosch, The Netherlands) as per standard protocol. Data were collected and analyzed as previously described.^16^ Phosphorylation data were collected by serial images captured using the PamGene Evolve Software on the PamStation®12 platform over multiple computer‐controlled pumping cycles and exposure times (10, 20, 50, 100, 200 ms). Raw image analysis was conducted using Evolve2, and comparative analysis and upstream kinase prediction were done in BioNavigator v6.2 using scoring from Kinexus (Kinexus Bioinformatics Corporation, Vancouver, Canada). In addition to kinetic reads, postwash captures were taken at 10, 20, 50, 100, and 200 ms exposures. These values were integrated into a slope, multiplied by 100 and log2 transformed.

Whole chip comparative analysis (BioNavigator Upkin PTK v14.0 and STK v 8.0) was conducted to identify the kinases most highly implicated by the observed phosphorylation patterns. Kinases were ranked by median final score (FKS, the combined extent that targets of that kinase were altered) and kinase statistic (KSTAT, the specificity of the peptides altered for the indicated kinase). Kinases with absolute KSTAT>0.5 and FKS >2 were selected as the most activated kinases. For network analysis of kinases, these kinases were uploaded and network mapped using GeneGo MetaCore (http://portal.genego.com, Thompson Reuters) using a “Shortest Paths Network Model.”

For network analysis of identified kinases combined with proteins identified in dACM from the Kiloplex array, information was input into STRING V10.5^18^ and all evidence sources for interactions were used with a minimum required interaction score of 0.7.

#### *Statistics*

For histological analysis, HMVEC proliferation, and HMVEC migration experiments, statistical analysis was conducted using a one‐way ANOVA with a post hoc Bonferroni test, with *p* < 0.05 considered significant (Prism, GraphPad Software, San Diego, CA). For both in vivo and in vitro gene expression and protein production experiments an unpaired Student's t test was conducted, with *p* < 0.05 considered significant (Prism, GraphPad Software). Throughout the manuscript, *p*‐value levels are defined as follows unless otherwise specified: * denotes *p* < 0.05, ** denotes *p* < 0.01, and ◊ denotes *p* < 0.001.

## RESULTS

**Figure 1:**
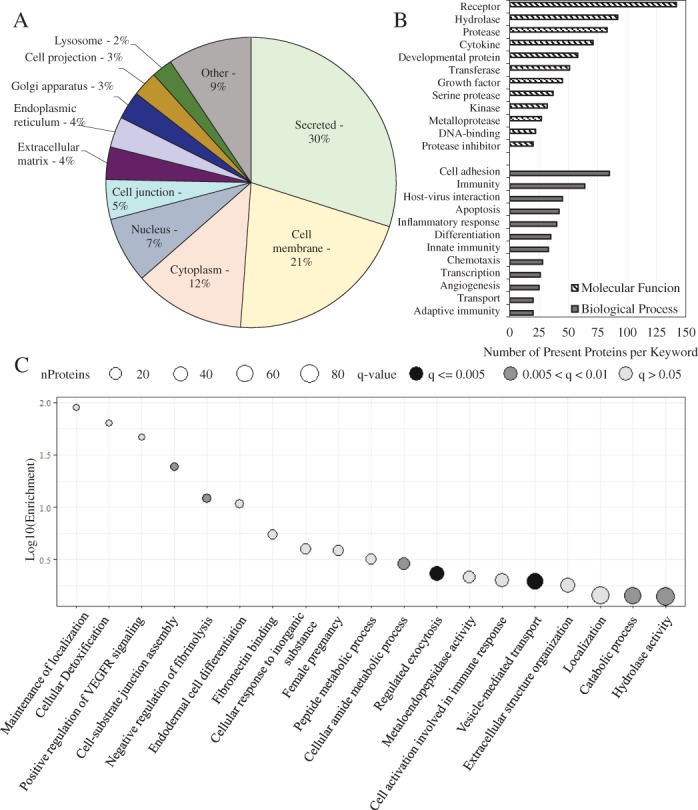
Most highly represented Uniprot keywords for cellular component (A) or biological process and molecular function (B) subclasses. (C) Enriched biological process and molecular function GO terms based on protein abundance.

Of the 1,000 proteins and growth factors evaluated in the kiloplex array, 640 were, on average, above the level of detection across the 10 donors tested. Proteins detected in dACM have diverse functional roles and localization (Figure 1A and B). Of the 640 proteins detected, approximately 30% were secreted, 21% were cell membrane related, 12% were cytoplasmic, 7% were nuclear, 5% were cell junction, and 4% were extracellular matrix proteins (Figure 1A). Receptors, hydrolases, proteases, and cytokines were the most commonly tagged molecular function terms, with growth factors, kinases, metalloproteases and protease inhibitors also present. Cell adhesion and immunity were the most highly represented biological process terms of interest, with inflammatory response, chemotaxis, and angiogenesis also represented (Figure 1B). The third biological process represented was host–virus interactions; these consisted of a number of integrins and other receptors that serve as viral receptors and proteins related to the modulation of immunity that occurs during pregnancy.^19^ Unbiased gene set enrichment analysis implicated cell‐substrate junction assembly, vascular endothelial growth receptor signaling, and endodermal cell differentiation as some of the most significantly enriched gene sets from the most abundant proteins (Figure 1C).

**Figure 2:**
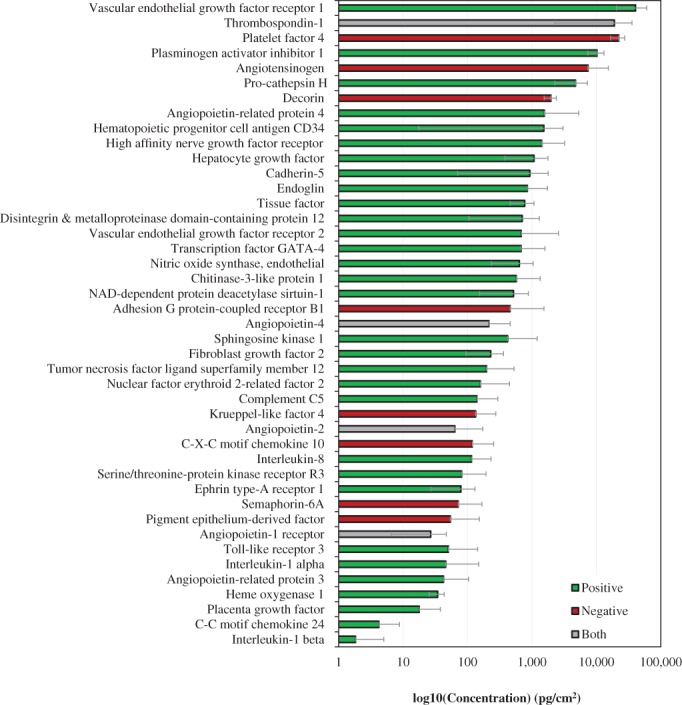
Protein levels of positive and negative regulators of angiogenesis based on GO annotation. Bars are average across all donors and are colored according to direction of angiogenic modulation: green are positive regulators (GO:0045766), red are negative regulators (GO:0016525), and gray are both positive and negative. Error bars are standard deviation.

**Figure 3:**
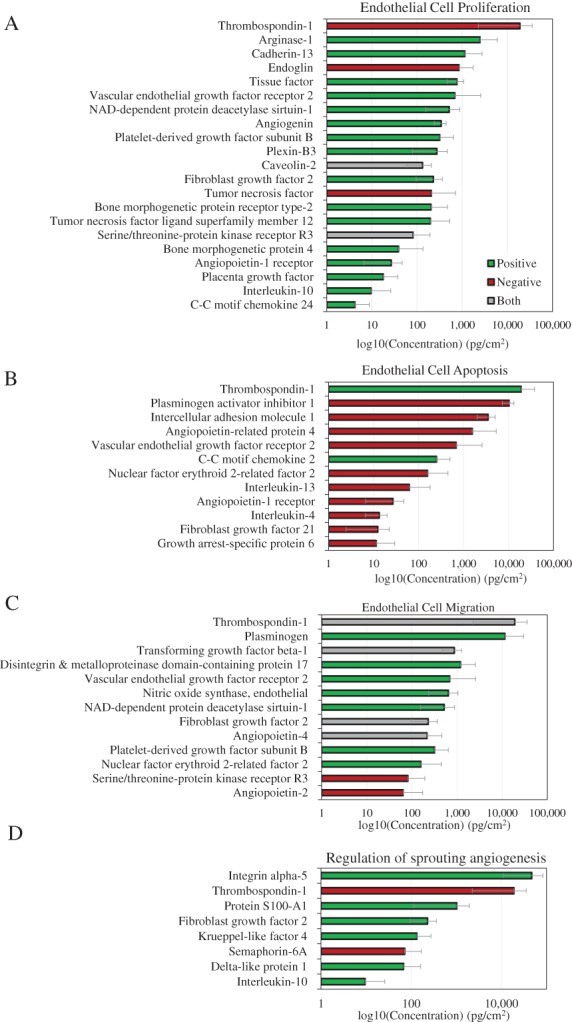
Angiogenic subclasses classes and protein levels based on GO annotations. Bars are average across all donors and are colored according to direction of angiogenic modulation: green are positive regulators, red are negative regulators, and gray are both positive and negative. Error bars are standard deviation. (A) Endothelial cell proliferation (GO:0001938 and GO:0001937). (B) Endothelial cell apoptotic process (GO:2000353 and GO:2000352). (C) Endothelial cell migration (GO:0043536 and GO:0043547). (D) Sprouting angiogenesis (GO:1903672 and GO:1903671).

Based on the significant body of work suggesting placental membranes support and promote angiogenic responses in vitro,^10^, ^11^, ^12^ using the kiloplex platform we then identified proteins found within dACM known to play a role in the regulation of angiogenesis (Figure 2). Of the 43 angiogenesis‐related proteins detected, 72% were pro‐angiogenic, 19% were anti‐angiogenic, and 9% had a dual function. To further elucidate, we identified dACM proteins annotated as regulators of specific angiogenic subprocesses, including endothelial cell proliferation and apoptosis, endothelial cell migration, and sprouting angiogenesis (Figure 3A–D). Of the 21 endothelial cell proliferation‐related proteins, 16 were tagged as promoters of proliferation, while 3 were tagged as inhibitors of proliferation. Additionally, 10 of the 12 proteins were tagged as negative regulators of endothelial cell apoptosis, while 2 proteins were positive regulators of apoptosis. Similarly, 7 of the 13 proteins tagged as regulators of endothelial cell migration were positive regulators, 2 were negative regulators and 4 were proteins labeled as having a dual function. Finally, 6 of the 8 proteins were labeled as positive regulators of sprouting angiogenesis while the other 2 were labeled as negative regulators.

**Figure 4:**
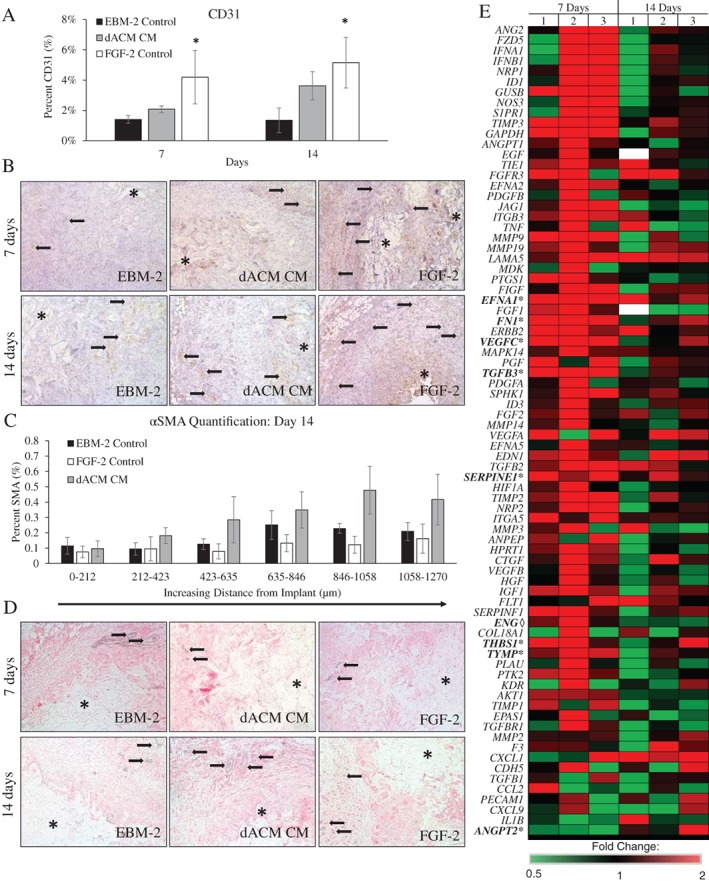
dACM CM induces an angiogenic responses in vivo. (A) CD31 quantification from implants retrieved at 7 and 14 days with representative images (B) where * indicates the implant and black arrows highlights vessels. (C) αSMA quantification from implants retrieved at 7 and 14 days with representative images (D) where * indicates the implant and black arrows highlight vessels. (E) Heat map of relative fold change of gene expression for dACM CM implants at 7‐ and 14‐days post‐implant (*n* = 3). Here green values represent down regulation, black values represent no change, and red values represent upregulation of respective targets. White boxes represent no reported value. Targets with significant differences in expression from controls are shown in bold where * indicates significance at day 7 and ◊ represents significance at day 14.

Histological evaluation of retrieved gel foam implants indicated increased angiogenesis as determined by CD31 and αSMA staining (Figure 4). Although not significant, at both 7 and 14 days CD31 staining was elevated along the periphery of the implant for dACM CM implants compared to the EBM‐2 control (Figure 4A and B). At 7 days, no differences in αSMA staining was observed between control and dACM CM groups along the periphery of the implant (Figure 4C). However, by 14 days αSMA was slightly increased in dACM CM groups compared to controls beginning approximately 200 μM from the edge of the implant (Figure 4C and D). Gene expression analysis of retrieved gelfoam inserts revealed significant changes in the expression of several targets in dACM‐treated versus control implants (Figure 4E). Specifically, at 7 days, the dACM‐treated group had significantly increased expression of the pro‐angiogenic genes including: fibronectin (*FN1*, 3.17 ± 0.38, average ± standard deviation, *n* = 3), ephrin A1 (*EFNA1*, 3.59 ± 0.73), TGF‐β3 (*TGFB3*, 2.94 ± 0.42), vascular endothelial growth factor C (*VEGFC*, 3.14 ± 0.93), thymidine phosphorylase (*TYMP*, 1.74 ± 0.23), thrombospondin 1 (*THBS1*, 1.78 ± 0.46), and serpin family E member 1 (*SERPINE1*, 2.22 ± 0.61). Angiopoietin‐2 (*ANGPT2*, 0.52 ± 0.18), an antagonist of angiopoietin 1, was significantly downregulated at 7 days. By 14 days only endoglin (*ENG*, 0.74 ± 0.05) was found to be significantly downregulated compared to controls.

**Figure 5:**
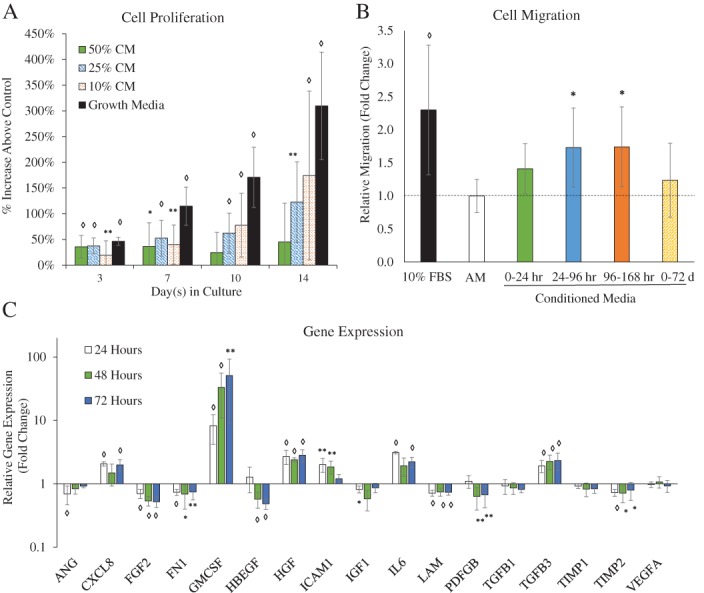
dACM CM induces an angiogenic responses in vitro. (A) quantitative proliferation assay in assay media alone or dACM CM in assay media (with 50%, 25%, and 10% CM by volume, *n* = 17); (B) quantitative analysis of HMVEC migration in assay media, growth media, or dACM CM (*n* = 16); (C) gene expression changes (at 24, 48, and 72 hours) in dACM (relative to assay media) with *GAPDH* as the internal control (*n* = 9) Average ± standard deviation, significance compared to assay media is denoted by: * denotes *p* < 0.05, ** denotes *p* < 0.01, and ◊ denotes *p* < 0.001.

When evaluating the effects of dACM CM in vitro, we observed significant changes in HMVEC proliferation, migration, gene expression, and protein production (Figure 5). Significant changes in cell proliferation were observed beginning at day 3 and continuing through day 14 (Figure 5A), at which point both 25% and 10% CM groups had significantly higher rates of proliferation at 122% ± 87% and 174% ± 168% relative to controls (average ± standard deviation, *n* = 17, *p* < 0.01). HMVEC migration was also significantly increased in dACM‐treated cells relative to assay media controls. Of note, we observed that HMVEC migration was dependent on the time‐period for CM collection; CM collected from 24 to 96 hours and 96 to 168 hours resulted in a significant increase in HMVEC migration (Figure 5B). These results are consistent with prior reports of placental membranes inducing endothelial cell proliferation and migration in vitro.^10^, ^11^, ^12^ Exposure of HMVECs to dACM CM induced significant changes in the expression of several genes (Figure 5C). Genes encoding for granulocyte‐macrophage colony‐stimulating factor (*GM‐CSF*), hepatocyte growth factor (HGF), and transforming growth factor beta‐3 (*TGFB3*) were significantly upregulated at all three time points compared to controls, whereas genes encoding for basic fibroblast growth factor (*FGF2*), fibronectin (*FN1*), laminin (*LAM*), and tissue inhibitors of metalloproteinase 2 (*TIMP2*) were downregulated at all three time points.

**Figure 6:**
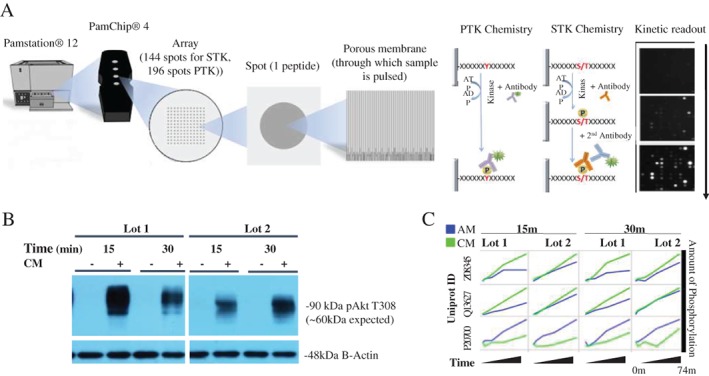
(A) Overview of kinomics platform, adapted with permission from PamGene International B.V. (https://www.pamgene.com/upload/image/brochures/Kinase%20brochure%2020180314%20vRL.pdf) (B) AKT t308 Western blot for timepoint selection showing broad activation in cells treated with dACM CM. (C) Kinetic phosphorylation plots for three representative peptide probes.

**Figure 7:**
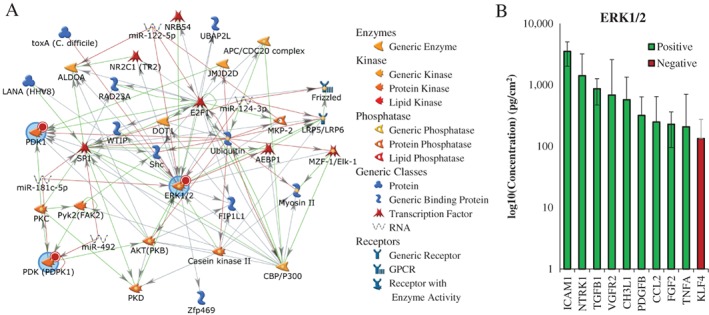
(A) Inferred protein network (MetaCore) from activated kinases (blue circles). Network was auto‐expanded to a maximum of 50 nodes. Red circles indicate query kinases increased in CM treated cells. Arrow heads denote direction of literature‐annotated interactions with color of line indicating type (red is negative, green is positive, gray is complex or unknown). MetaCore symbol legend on right. (B) Protein levels of positive and negative regulators of the ERK1/2 cascade based on GO annotation. Bars are average across all donors and are colored according to direction of angiogenic modulation: green are positive regulators (GO:0070374), red are negative regulators (GO:0070373). Error bars are standard deviation.

We then performed a global analysis of both serine/threonine and tyrosine kinase activity using a commercial platform (PamChip, PamGene, the Netherlands) to characterize intracellular signaling pathways activated by dACM in vitro. This platform directly detects phosphorylation of ~290 peptides in a kinetic, high‐throughput assay (Figure 6A). Kinase activity was measured in lysates from HMVECs exposed to dACM CM for 15 and 30 minutes; determination of timepoints was based on western blots showing clear phosphorylation of phospho‐Akt at these timepoints (Figure 6B). Representative dynamic phosphorylation graphs for three peptide probes can be found in Figure 6C. dACM‐derived factors were found to drive gross changes in kinase activity, with whole‐chip analysis revealing mitogen‐activated protein kinase 3 (MAPK3/ERK1), mitogen‐activated protein kinase 1 (MAPK1/ERK2), inactive serine/threonine‐protein kinase (TEX14), pyruvate dehydrogenase (acetyl‐transferring) kinase isozyme 1, mitochondrial (PDK1), and 3‐phosphoinositide‐dependent protein kinase 1 (PDPK1) as the kinases most active in dACM groups compared to control (based on strength and specificity scores, Supporting Information Table S2). Protein‐network analysis of these five kinases revealed an ERK1/2 centric network (Figure 7A). Putative dACM proteins potentially responsible for ERK‐activation were subsequently identified by searching the Kiloplex data for dACM proteins annotated as positive and negative regulators of the ERK pathway. Of the 10 detected dACM proteins related to ERK1/2 signaling, 9 are positive regulators and only 1 is a negative regulator of ERK (Figure 7B). Of those 10 proteins, high affinity nerve growth factor receptor (NTRK1), VGFR2, chitinase‐3‐like protein 1 (CH3L1), and FGF2 are positive regulators of angiogenesis, while Krueppel‐like factor 4 (KLF4)—the lone ERK1/2 antagonist—is a negative regulator of angiogenesis.

**Figure 8:**
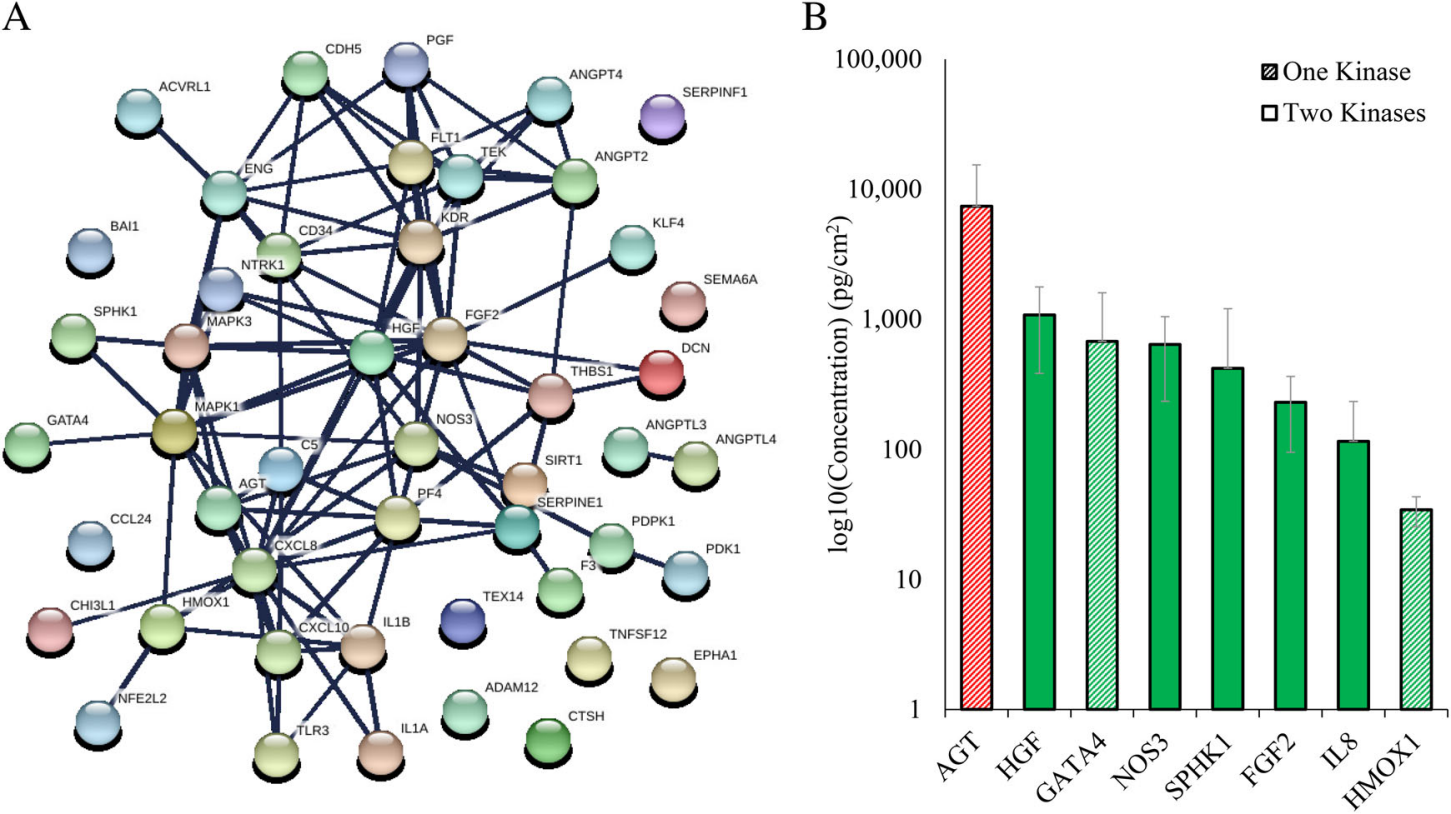
Inferred networks of activated kinases with angiogenesis‐related proteins detected in Kiloplex array. (A) Inferred network (STRING). Disconnected nodes are hidden, node colors and spacing are arbitrary. (B) Levels of angiogenic proteins directly connected with the five most activated kinases based on STRING network in A. Bars are average across all donors, are hashed according to the number of kinases to which they were connected, and are colored according to direction of angiogenic modulation: green are positive regulators (GO:0070374), red are negative regulators (GO:0070373). Error bars are standard deviation.

To identify angiogenesis‐related proteins potentially responsible for the observed dACM‐induced kinase activation, we interrogated the protein‐interaction network between our 5 activated kinases and the 43 angiogenesis‐related proteins identified to be present in dACM from the Kiloplex array (Figure 8). Any proteins with a direct network connection to one of the 5 activated kinases were graphed with concentrations (Figure 8B). Of these eight proteins, all are classified as positive regulators of angiogenesis except for angiotensinogen (ANGT), which is classified as a negative regulator. With the exception of nitric oxide synthase endothelial (NOS3), which is connected to ERK1/MAPK3 and PDPK1, all other proteins connected to two kinases were connected to ERK1/MAPK3 and ERK2/MAPK1. Two of the six activated kinases, PDK1 and TEX14, had no direct connections to any angiogenesis‐related proteins detected in the Kiloplex array. The most abundant proteins implicated from this analysis were AGT, hepatocyte growth factor (HGF), and Transcription factor GATA‐4 (GATA4). One of the proteins implicated from this analysis—FGF2—was also implicated as positive regulators of ERK1/2 signaling (Figure 7B).

## DISCUSSION

Angiogenesis is an essential part of tissue repair, and the failure to establish new vasculature following injury is thought to stall the healing process. Consequently, this study focused on better understanding how placental membranes, specifically dACM, promote angiogenesis in vitro and in vivo. First, we completed an in‐depth characterization of the proteins found within these membranes. While prior work has focused on specific subsets of growth factors and cytokines in these tissues,^10^, ^20^ one goal of this work was to attempt to create a more complete proteomic profile of this tissue. Although many of the observed proteins may not have a significant effect on angiogenesis, a comprehensive understanding of dACM will allow for a more complete understanding of the effects seen here and in prior *in vitro* studies.^20^ In this study, we observed a mix of both angiogenic promoters and inhibitors; this combination is important and expected as angiogenesis is a well‐organized and spatiotemporally controlled process which utilizes both promoters and inhibitors to establish an effective stable network of blood vessels.^2^, ^3^ In general, the majority of angiogenic‐related proteins measured in dACM were promoters of angiogenesis. Of interest, we observed that VEGFr1 and TSP1 were the most abundant angiogenesis‐related proteins. VEGFr1 is a positive regulator of angiogenesis, with corresponding pro‐angiogenic classifications of proliferation, apoptosis, and migration subprocesses.^15^ TSP‐1 is classed as both a positive and negative regulator of angiogenesis. The role of TSP1 in angiogenesis is highly complex and has been the subject of extensive review.^21^, ^22^, ^23^, ^24^, ^25^ Since its discovery as one of first anti‐angiogenic proteins, TSP‐1 has been shown to promote angiogenesis in a context‐dependent manner. Pro‐angiogenic phenotypes of endothelial cells such a proliferation, migration, and adherence depend on various factors including cell–cell contact, extracellular processing, matrix and receptor protein localization and signaling, and TSP1 levels.^26^, ^27^, ^28^, ^29^ In the context of dACM, the overall effect of the graft on HUVECs was pro‐angiogenic, suggesting that the TSP1 present either functions to promote angiogenesis or has anti‐angiogenic effects that are outweighed by the remaining pro‐angiogenic protein content of the graft. It is indeed clear that the majority of proliferation, migration, and sprouting‐related proteins were activating, and the majority of apoptosis‐related proteins were negative regulators.

The results from the in vivo study further supported our hypothesis, where the releasate from dACM was found to promote angiogenesis in vivo. The increased expression of pro‐angiogenic genes 7 days post implant indicates that the growth factors and cytokines present within the dACM CM have extended effects. Prior work has shown that amnion/chorion membranes can promote angiogenesis in vivo^10^; however, to the best of our knowledge, this is the first study to show that the dACM releasate alone can promote similar effects.

The in vitro results provide insight into potential ways dACM mediates the angiogenic responses observed in vivo. Endothelial cell proliferation and migration are two important steps early in angiogenesis; the increases in migration and proliferation observed here are likely due to a combination of factors present in dACM, as indicated in Figure 3. Additionally, dACM CM exposure resulted in increased GM‐CSF expression. GM‐CSF has been shown to improve angiogenesis in both acute and chronic wounds.^30^ When comparing these results to prior studies, the upregulation of both GM‐CSF and TGF‐β3 seen in our study is consistent with prior studies, while the downregulation of PDGF‐BB contradicts the findings of earlier studies evaluating endothelial cell response with amnion/chorion membranes.^10^ Differences may be due to different methodologies in graft processing or how CM from the grafts were obtained. When considering both in vitro and in vivo models, TGFβ3 was consistently upregulated in response to dACM CM. While the cause of this upregulation is unclear, TGF‐β3 has been associated with improved wound repair outcomes and reduced scarring.^31^, ^32^ Another interesting observation was that FN1 was upregulated in vivo but not in vitro. This difference may be due to difference in timepoints or because the in vitro model evaluated a single‐cell type in a two‐dimensional environment.

We also utilized a kinomics platform to determine how dACM CM alters intracellular signaling within endothelial cells in vitro. Kinase‐driven signal transduction cascades are critical in almost every aspect of cell biology, and with more than 450 of 518 kinases being linked to disease progression, kinases have gained considerable attention as therapeutic targets.^17^ As such, the majority of large‐scale kinomics platforms have been primarily used for kinase drug discovery applications.^16^, ^17^, ^33^ While kinomic platforms are increasingly being used to interrogate signaling pathways and basic biology in other applications, this technique has not been widely applied to studying tissue repair. Kinomic analysis implicated ERK1/2, PDPK1, PDK1, and TEX14 as the kinases most significantly activated in endothelial cells by dACM in vitro. Network modeling of dACM activated kinases with angiogenic proteins identified from the Kiloplex array elucidated proteins found within these tissues that may be responsible for the observed pro‐angiogenic phenotypes, including angiotensinogen (ANGT, connected to ERK2) and hepatocyte growth factor (HGF, connected to ERK1/2). ANGT is annotated as an anti‐angiogenic protein; however, ANGT is a precursor to angiotensin II (ANGII), which has been shown to stimulate proliferation of HUVECs through a VEGF‐dependent mechanism.^34^ Further, in cultured cardiac microvascular endothelial cells, ANGII was found to increase or decrease tubulogenesis in both time and dose‐dependent manners through ERK phosphorylation.^35^

HGF is a potent pro‐angiogenic factor, which has been shown to have multiple effects on endothelial cells including the induction of proliferation and migration, inhibiting TGF‐β1 secretion, and attenuating cellular senescence induced by angiotensin II; in smooth muscle cells HGF has been shown to upregulate the production of VEGFA.^36^ Additionally, HGF has been implicated in a signaling pathway necessary for branching morphogenesis of Madin–Darby canine kidney (MDCK) cells.^37^ In MDKC cells, HGF‐initiated signal transduction through ERK ultimately led to increased levels of fibronectin, previously demonstrated to be required for tubulogenesis in a variety of systems.^37^ In the current study, dACM treatment resulted in increased FN1 expression in vivo.

Network analysis of Kiloplex‐detected dACM proteins involved in the ERK1/2 signaling pathway revealed pro‐angiogenic proteins including TGFβ1, chitinase‐3‐like protein (CH3L1), and FGF2. Interestingly, in spite of the presence of TGFβ1 in dACM and the observed upregulation of TGFβ3 in this study, we did not observe significant changes in downstream SMAD or ALK5/ALK1 activity in endothelial cells in vitro. This may be due to the timeframe in which this kinase activity was measured or the single‐cell type evaluated. The importance of FGF2 in angiogenesis has been well established.^38^ In one study of FGF‐2 deficient mouse endothelial cells, FGF‐2 activation of ERK signaling was required for cell migration.^39^ Comparatively little work has been done on CH3L1 (also known as YKL‐40, a heparin‐binding glycoprotein), but due to its role in cancer, CH3L1 has been characterized in the context of tumor angiogenesis. Recombinant CH3L1 has been found to promote migration, tube formation, and (to a lesser extent) proliferation of HMVECs, with CH3L1‐stimulated tube formation abrogated by inhibition of ERK1/2.^40^

Taken together, these results emphasize that the angiogenic effects observed both in vitro and in vivo are likely due to the synergistic activity of multiple growth factors and cytokines. This work identified several angiogenic growth factors known to be important in placental tissues, including angiogenin, angiopoietin‐2, EFG, BFGF, HB‐EGF, HGF, PDGF‐BB, PIGF, and VEGF.^10^, ^11^, ^12^ Additionally, by evaluating this data using an unbiased approach, we have also identified a number of growth factors and cytokines that previously have been unstudied in this context including CH3L1.

While not the focus of this work, it is important to note the ECM makeup of dACM, as proteins and growth factors studied in this work directly interact with matrix proteins. Additionally, the ECM makeup may affect the release of growth factors and cytokines from this tissue. Prior characterization of dACM found that the ECM was primarily composed of collagen, including collagens I and III, with a significant amount of sulfated glycosaminoglycans, hyaluronic acid, and elastin. In the same study, immunohistochemical staining identified other ECM proteins present in dACM, including fibronectin and laminin.^20^ In contrast to dermis, collagen, and synthetic polymer matrices, dACM ECM generally has a higher glycoprotein content including both sulfated glycosaminoglycans and hyaluronic acid relative to the collagen content.^41^

While an in vivo model was used to establish the pro‐angiogenic effects of dACM CM, the majority of this work was conducted in vitro on human microvascular dermal endothelial cells. One limitation of this approach is that it does not take into account the three‐dimensional environment and complex multicellular systems of human tissue. Additionally, this study focused on the effects of the releasate from dACM and did not evaluate the graft as a whole. While this is important, because it indicates that proteins released from dACM are bioactive in nature; it does not take into account the dynamic release of proteins over time or the potential effects of the extracellular matrix. Lastly, protein‐interaction networks were based on the Kiloplex array, which measured levels of 1,000 proteins in dACM graft extract. However, in models throughout this study, the releasate (or CM) from these grafts was evaluated. While prior work has evaluated the release rates of 25 growth factors and cytokines from these grafts and determined that on average 94% of proteins are released from the graft within 7 days,^20^ specific release characteristics are target dependent. As such, it is possible that some proteins included in the network analysis may not be present in graft releasate used for these experiments. Future in vitro work will focus on dACM grafts as a whole and measuring their impact in more complex models that better represent complex multicellular three‐dimensional environments. It also may be valuable to utilize the kinomics platform for in vivo studies to better characterize the effect of dACM on intracellular signaling within the tissue as a whole.

## Source of funding

This study was supported and funded by Organogenesis, Canton, MA.

## Conflict of Interest

JPM, MB, KAK, MK and KCM are employees of Organogenesis.

## Authors Contributions

JM: designed study, completed experiments, analyzed data, and wrote manuscript; MB: designed study, completed experiments, analyzed data, and wrote manuscript; MK: completed experiments and analyzed data; KK: completed experiments and analyzed data; KM: designed study, reviewed the data, wrote manuscript, and edited manuscript. All authors have reviewed and approved the final version of the manuscript

## Supporting information


**Supplemental Table 1.** Taqman probes used for quantitative PCR.
**Supplemental Table 2.** Summary of most significantly activated kinases in dACM‐treated versus control groups.
