# Supplementary material for: A mechanistic evaluation of the angiogenic properties of a dehydrated amnion chorion membrane in vitro and in vivo
Source: Wound Repair Regen. 2019 Aug 29;27(6):609–21. doi: 10.1111/wrr.12757 (PMC6900065; doi:10.1111/wrr.12757)
Supplement: Supplementary file 1 — Supplemental Table 1. Taqman probes used for quantitative PCR. Supplemental Table 2. Summary of most significantly activated kinases in dACM‐treated versus control groups. [file WRR-27-609-s001.docx]

**Tables**

**Supplemental Table 1:** Taqman probes used for quantitative PCR.

| **Cytokine Name** | **Gene Abbreviation** | **Ascension Number** |
| --- | --- | --- |
| Angiogenin | ANG | Hs04195574_sH |
| Interleukin 8 | CXCL8 | Hs00174103_m1 |
| Basic Fibroblast Growth Factor | FGF2 | Hs00266645_m1 |
| Fibronectin | FN1 | Hs01549976_m1 |
| Granulocyte Macrophage Colony Stimulating Factor | GM-CSF | Hs00929873_m1 |
| Heparin Binding EGF-like Growth Factor | HBEGF | Hs00181813_m1 |
| Hepatocyte Growth Factor | HGF | Hs00300159_m1 |
| Intercellular Adhesion Molecule 1 | ICAM1 | Hs00164932_m1 |
| Insulin-like Growth Factor I | IGF-I | Hs01547656_m1 |
| Interleukin 6 | IL6 | Hs00174131_m1 |
| Laminin | LAMA4 | Hs00935293_m1 |
| Platelet Derived Growth Factor BB (PDGF-BB) | PDGFB | Hs00966522_m1 |
| Transforming growth factor beta 1 | TGFB1 | Hs00998133_m1 |
| Transforming growth factor beta 3 | TGFB3 | Hs01086000_m1 |
| TIMP metallopeptidase inhibitor 1 | TIMP1 | Hs01092512_g1 |
| TIMP metallopeptidase inhibitor 2 | TIMP2 | Hs00234278_m1 |
| Vascular endothelial growth factor A | VEGF | Hs00900055_m1 |

**Supplemental Table 2:** Summary of most significantly activated kinases in dACM-treated versus control groups.

| **Kinase Name** | **Kinase Uniprot ID** | **Mean Final Score** | **Mean Kinase Statistic** | **Uniprot Description** [provided by UniProtKB Feb. 2019] |
| --- | --- | --- | --- | --- |
| ERK1 | P27361 | 3.19 | -0.52 | Serine/threonine kinase which acts as an essential component of the MAP kinase signal transduction pathway. MAPK1/ERK2 and MAPK3/ERK1 are the 2 MAPKs which play an important role in the MAPK/ERK cascade. Depending on the cellular context, the MAPK/ERK cascade mediates diverse biological functions such as cell growth, adhesion, survival and differentiation through the regulation of transcription, translation, cytoskeletal rearrangements. |
| TEX14 | Q8IWB6 | 3.10 | -0.73 | Required both for the formation of intercellular bridges during meiosis and for kinetochore-microtubule attachment during mitosis. During mitosis TEX14 is recruited to kinetochores by PLK1 during early mitosis and regulates the maturation of the outer kinetochores and microtubule attachment. Has no protein kinase activity *in vitro* (By similarity). |
| ERK2 | P28482 | 2.93 | -0.50 | Serine/threonine kinase which acts as an essential component of the MAP kinase signal transduction pathway. MAPK1/ERK2 and MAPK3/ERK1 are the 2 MAPKs which play an important role in the MAPK/ERK cascade. Depending on the cellular context, the MAPK/ERK cascade mediates diverse biological functions such as cell growth, adhesion, survival and differentiation through the regulation of transcription, translation, cytoskeletal rearrangements. |
| PDPK1 | O15530 | 2.92 | -0.98 | Serine/threonine kinase which acts as a master kinase, phosphorylating and activating a subgroup of the AGC family of protein kinases. Plays a central role in the transduction of signals from insulin by providing the activating phosphorylation to PKB/AKT1, thus propagating the signal to downstream targets controlling cell proliferation and survival. Negatively regulates the TGF-beta-induced signaling by: modulating the association of SMAD3 and SMAD7 with TGF-beta receptor, phosphorylating SMAD2, SMAD3, SMAD4 and SMAD7, preventing the nuclear translocation of SMAD3 and SMAD4 and the translocation of SMAD7 from the nucleus to the cytoplasm in response to TGF-beta. Activates the NF-kappa-B pathway via phosphorylation of IKKB. The tyrosine phosphorylated form is crucial for the regulation of focal adhesions by angiotensin II. Essential for the motility of vascular endothelial cells (ECs) and is involved in the regulation of their chemotaxis. |
| PDK1 | Q15118 | 2.43 | -0.86 | Kinase that plays a key role in regulation of glucose and fatty acid metabolism and homeostasis via phosphorylation of the pyruvate dehydrogenase subunits PDHA1 and PDHA2. Plays an important role in cellular responses to hypoxia and is important for cell proliferation under hypoxia. Protects cells against apoptosis in response to hypoxia and oxidative stress. |
